# Both MicroRNA-155 and Virus-Encoded MiR-155 Ortholog Regulate TLR3 Expression

**DOI:** 10.1371/journal.pone.0126012

**Published:** 2015-05-04

**Authors:** Xuming Hu, Jianqiang Ye, Aijian Qin, Haitao Zou, Hongxia Shao, Kun Qian

**Affiliations:** 1 Ministry of Education Key Lab for Avian Preventive Medicine, Yangzhou University, Yangzhou, 225009, P.R. China; 2 Key Laboratory of Jiangsu Preventive Veterinary Medicine, Yangzhou University, Yangzhou, 225009, P.R. China; 3 Jiangsu Co-innovation Center for Prevention and Control of Important Animal Infectious Diseases and Zoonoses, Yangzhou, 225009, P.R. China; H.Lee Moffitt Cancer Center & Research Institute, UNITED STATES

## Abstract

MicroRNA-155 (miR-155) has been as an important controller of TLR3 signalling. However, the interactions between miR-155 and TLR3 are poorly understood. Here, we focused on the regulation of the relationship between miR-155 and TLR3. Sequence analyses and firefly luciferase reporter assay revealed that miR-155 target were present in the coding sequences (CDS) of TLR3. And the expression of the TLR3 protein could be inhibited by a miR-155 mimic or by a virally encoded orthologue in chick embryo fibroblast cells. Notably, endogenous miR-155 induction emerged a negative regulation on TLR3 expression in TLR2, 4 and 7 ligands stimulated HD11 cells, an avian macrophage cell line. Moreover, treatment with the miR-155 antagomir increased TLR3 levels while significantly decreased the abundance of TLR3 with miR-155 agomir. In addition, our data showed that miR-155 could inhibit IFN-β production possibly though TLR3 signal pathway. All these findings might reveal a new mechanism by which miR-155 can regulate the TLR3 immune response.

## Introduction

Toll-like receptor (TLR) has key roles in the recognition of pathogens and the initiation of the innate immune response that subsequently primes the specific adaptive immune response during infection [1, 2]. The activation of TLR not only has implications for antiviral defence but also has contributions for tumour suppression including apoptosis and anti-angiogenesis [3–5]. MicroRNAs are small noncoding RNAs that regulate gene expression at the post-transcriptional level and have been as a critical regulatory factor in the mammalian immune system [6, 7]. Recently, microRNAs has been documented to play vital roles in TLR immunity and been as the fine-tuners of TLR signalling [8, 9]. For example, the members of the let-7 microRNA family [10] and miR-105 can regulate mRNA level of TLR4 and TLR2 respectively [11]. Moreover, the activation of TLR signals can also induce microRNAs (such as miR-146, miR-155 and miR-223) that can feedback the components in the TLR signalling system[9, 10]. However, little is known about the roles of individual microRNAs in the TLR3 immune response.

One microRNA, miR-155 is an ancient regulator of the immune system[12] and is essential for normal immune function and antibody production[13, 14]. Recently, miR-155 has been found to play vital roles in the regulation of the immune response. miR-155 also plays important roles in controlling the differentiation of CD4+T cells into helper T cells[14, 15] and the development of regulatory T cells[16, 17]. Moreover, miR-155 activates the response of CD8+ T cells to viral infection, and this activation is regulated by type I interferon signalling [18–20]. More recently, miR-155 has been as an important controller of TLR3 signalling via the targeting of adaptor molecules, downstream regulators and cytokines, such as TAB2, IKK-ε and RIP[8, 9]. Notably, several oncogenic herpes viruses have evolved alternative strategies that either supply miR-155-like activities by encoding functional orthologous of miR-155 or induce the expression of cellular miR-155 in viral tumourigenesis [21–23]. Thus, we speculated that miR-155 might control the TLR3 immune response in many manners that could be exploited by the virus via the encoding of functional orthologues of miR-155 to manipulate the host immune response.

We hypothesised that if miR-155 can directly control TLR3 expression, a target site of miR-155 might exist in the *TLR3* gene. Unfortunately, no miR-155 seed sequence was found in the 3′ untranslated regions (3′UTRs) of the *TLR3* mRNA. Tay Y et al. showed that many naturally occurring microRNA targets exist in the amino acid coding sequences (CDS) of mRNAs[24]. This finding indicates that the complete analysis of the regulatory targets of miR-155 should be expanded to TLR3 coding regions. Here, we reported that miR-155 negatively regulates TLR3 expression.

## Materials and Methods

### Ethics statement

This study was performed in strict accordance with the recommendations in the Guide for the Care and Use of Laboratory Animals of the Yangzhou University. The protocol was approved by the Committee on the Ethics of Animal Experiments of the Yangzhou University (Licence Number: 06R015).

### Reagents

The TLR2 ligand ultrapure *E*. *coli 0111*:*B4* peptidoglycan (PGN-EB), the TLR3 ligand synthetic analogue of dsRNA poly (I:C) with a high molecular weight, the TLR4 ligand ultrapure *E*. *coli 0111*:*B4* lipopolysaccharide (LPS-EB) and the TLR7 ligand small synthetic antiviral molecule Imiquimod (R837) were all from InvivoGen.

### Cells

Primary chicken embryo fibroblast cells (CEFs) were prepared from 10-day-old specific-pathogen-free (SPF) embryos that were obtained from Merial Vital (Laboratory Animal Technology CO., Ltd., Beijing, China). Secondary CEF cells were seeded in 6-well plates in Dulbecco’s Modified Eagle’s medium (DMEM; Life Technologies/GIBCO, MD, USA) with 5% foetal bovine serum (FBS) at 37°C, 5% CO2 and 95% humidity.

HD11 cells, an avian macrophage cell line, was maintained in the Dulbecco’s modified eagles media (DMEM) containing 10% chicken serum, antibiotics (100 U penicillin/ml and 100Ag streptomycin/ml), and 1.5 mM L-glutamine at 41°C, 5% CO2, and 95% humidity.

### TLR stimulation

Secondary CEF cells or HD11 cells were seeded in 6-well plate and incubated for 16~24 h in medium containing selected TLR ligands. After incubation, supernatants were collected, clarified and stored, and cells were washed and used for additional tests.

### Oligonucleotide transfection

The gga-mir-155 and mdv1-mir-M4-5p mimics, gga-mir-155 inhibitors, gga-miR-155 antagomir and agomir were synthesised by Ribobio, and the mimic and inhibitor Ncontrol, the antagomir and agomir Ncontrol were from Ribobio. The antagomir and agomir are miRNA antagonists and agonists with chemical special modification, which can has higher stability and inhibitory effect and does not require transfection reagents in cell experiment.

Oligonucleotide transfection was performed with Lipofectamine RNAiMax transfection reagent (Invitrogen). Secondary CEF cells were seeded in 6-well plate and incubated for 16~24 h. Next, cells were transfected for 48 h with gga-miR-155 mimic and inhibitor. HD11 cells were seeded in 6-well plate and treated for 48 h with gga-miR-155 antagomir and agomir.

### RNA extraction and quantitative real-time PCR

Total RNA was extracted with the miRNeasy Mini Kit (QIAGEN), and mature microRNAs were reverse transcribed with the miScript II RT Kit (QIAGEN) and amplified using the miScript SYBR Green PCR Kit (QIAGEN). miScript Primer Assays (QIAGEN) were used for the gga-mir-155 (assay ID: MSC0003997), mdv1-mir-M4 (assay ID: MSC0003997), and gga-mir-21 (assay ID: MSC0003998) in 96-well plates (Applied Biosystems) on an ABI 7500 device (Applied Biosystems). Gene expressions were calculated relative to miR-U6. Real-time PCR was performed on TLR3 gene as previously reported[25], and the gene expression levels were normalised to the expression of chicken 18S mRNA.

### Vector constructs and luciferase assay

The pri-gga-mir-155 and scrambled sequences were synthesised and cloned into pGV-268 vectors to generate the pGV-268-gga-mir-155 and the pGV268-scrambled control. The amino acid coding sequence of the chicken TLR3 (867–1073), mutant-type, putative gga-mir-155 binding site was cloned downstream of a firefly luciferase cassette in a pGV-272 vector.

These plasmids (0.4 μ g per well) were transfected into HEK293T cells (4 × 10^5^ cells per well) in 24-well plates with Lipofectamine 2000 (Invitrogen) following the manufacturer’s instructions. The cells were co-transfected with 0.4 μ g of the luciferase constructs and 0.3 μ g of the Renilla luciferase plasmid and incubated for 48 h. The Dual-Glo Luciferase assay was performed according to the manufacturer’s protocol (Promega).

### Immunoblot

Cells (5 × 10^5^ to 10 ×10^5^) were lysed with Cell Lysis Buffer (10X) (Cell Signaling Technologies, 9803) with protease inhibitors. The samples were loaded with 5× denaturing sample buffer and separated by 12% SDS-PAGE. The proteins were transferred to polyvinylidene difluoride membranes and were subsequently analysed by immunoblot with the relevant antibodies. The blots were developed by using chemiluminescence (protein simple, Fluorchem E FE0605). The monoclonal antibody used to detect β-actin was from Santa Cruz (sc-47778), and the polyclonal antibody to TLR3 was from Novus Biologicals (NBP2-24565).

### Enzyme-linked immunosorbent assays

The cytokine concentrations in the supernatants were measured with an ELISA kits for chicken type I interferons IFN-α and IFN-β, pro-inflammatory cytokines, tumour necrosis factor-α (TNF-α), interleukin-6 (IL-6) and IL-1β according to the manufacturer’s instructions (from Shanghai Hengyuan Bioscience & Technology Company).

### Statistical analyses

Statistical analyses were performed with either the Statistical Package for the Social Sciences (version 16.0) or Excel GraphPad (Prism 5) software to determine the P-values by paired Student’s t-tests or unpaired tests for normal distributions of at least three independent experiments.

## Results

### Target site of miR-155 in the TLR3 coding region

**Fig 1:**
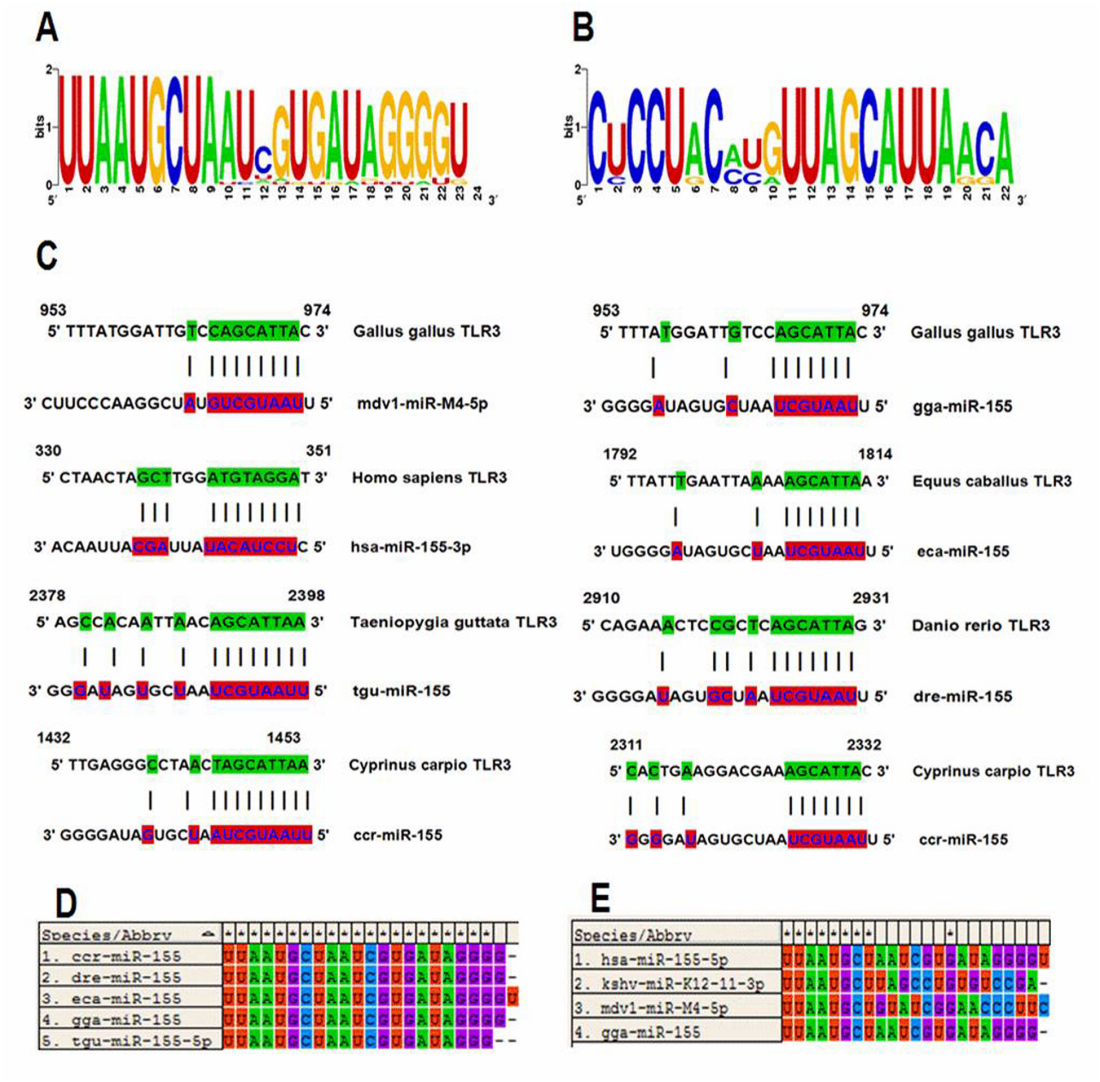
Analysis of the miR-155 seed sequence in the TLR3 coding region. (a and b) The sequence logo for miR-155 was generated by Weblogo (http://weblogo.berkeley.edu/). (c) The target sites of the miR-155 sequence in the TLR3 coding regions from Homo sapiens, Gallus gallus, Danio rerio, Equus caballus, Taeniopygia guttata and Cyprinus carpio at the seed matches of the 8mer and 7mer-m8 sites. (d and e) Clustal W analysis of the miR-155 sequence with Molecular Evolutionary Genetics Analysis version 5 (MEGA5) software.

The target sites of the miR-155 sequence in the coding region of the Homo sapiens, Gallus gallus, Danio rerio, Equus caballus, Taeniopygia guttata and Cyprinus carpio TLR3 mRNAs were analysed by computational analyses and blast analyses of the miR-155 target sites. We found that there are at least approximately 23 miR-155-5p sequences, 7 miR-155-3p sequences and 2 virus-encoded miR-155 orthologues in the miRBase v.20. The sequence logo for miR-155 is shown in Fig 1a–1e. Moreover, the miR-155 sequences in these species were highly conserved (Fig 1d). Additionally, the target site of mdv1-miR-M4-5p, which is the functional orthologue of miR-155 that is encoded by MDV, is in the TLR3 coding region of Gallus gallus. These targets sequences indicate that miR-155 might directly target the TLR3 protein-coding region.

### miR-155 targets TLR3

**Fig 2:**
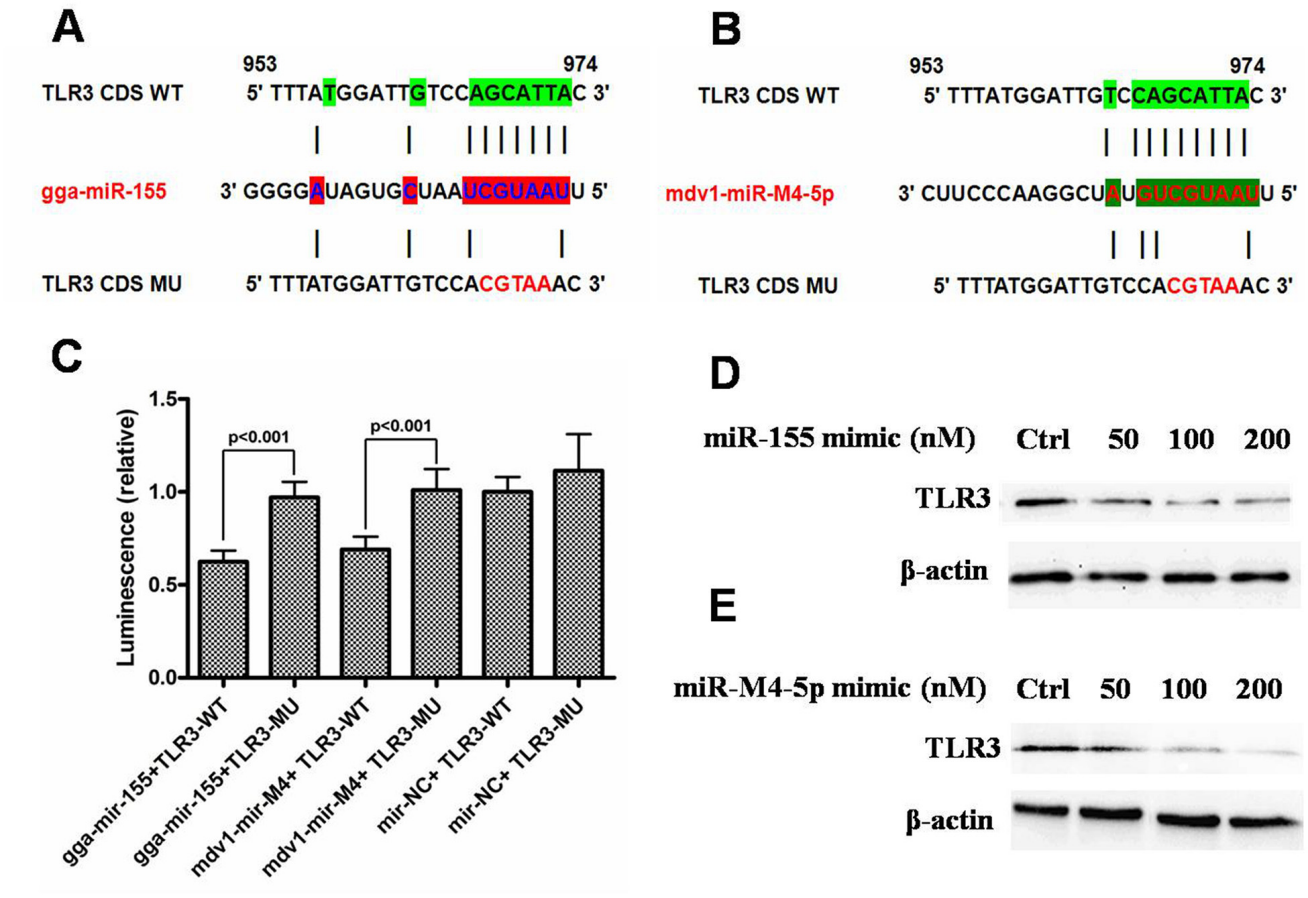
Luciferase reporter assay for the interaction between miR-155 and TLR3. (a and b) Predicted binding sites of gga-miR-155 and mdv1-mir-M4-5p in the coding region of the TLR3 mRNA. Five nucleotides were mutated in the coding region of the TLR3 mRNA. The numbers indicate the positions of the nucleotides in the wild-type sequences (NM_001011691.3). (c) Activity of the luciferase gene linked to the coding region of the TLR3 mRNA. The pGL3 firefly luciferase reporter plasmids with wild-type or mutant for the TLR3 mRNA coding regions were transiently transfected into HEK293 cells with the gga-miR-155 precursor, mdv1-mir-M4-5p or the negative control and a Renilla luciferase reporter for normalisation. Luciferase activities were measured after 48 hr. The data were presented as the means and the standard deviations (SDs) of separate transfections (n = 5). (d and e) The down-regulation of endogenous TLR3 protein expression by miR-155. CEF cells were transfected with 50–200 nM of the miR-155 mimic or the negative control mimic, and TLR3 expression in the cell lysates was analysed with western blotting. The expression of β-actin was used as a control.

We chose a pair of models of miR-155 (gga-miR-155 and mdv1-miR-M4-5p) to confirm the direct interaction between the TLR3 mRNA coding region and miR-155. We analysed the most likely target sites of gga-miR-155 and mdv1-miR-M4-5p in the coding region of TLR3 mRNA (Fig 2a and 2b) by focusing on the base pairing in the seed sequence of the miRNA because the seed sequence is considered to be a critical determiners in the recognition of target mRNA by miRNAs. We used normal sequences and sequences that were mutated at five nucleotides within the predicted seed binding sites of the coding region sequences of TLR3 mRNA and then performed luciferase reporter assays in HEK293T cells. Notably, we observed that gga-miR-155 significantly downregulated the expression of firefly luciferase fused to the TLR3 wild-type ORF, whereas mir-NC did not (Fig 2c). In the reciprocal experiment in which we transfected the cells with the firefly luciferase vector fused to the mutated TLR3 ORF, neither gga-miR-155 nor mir-NC affected the expression of firefly luciferase (Fig 2c). Additionally, the ability of mdv1-miR-M4 to bind the coding region of TLR3 was evaluated. The activity of the firefly luciferase fused to the TLR3 wild-type ORF was significantly suppressed by the wild-type mdv1-mir-M4; moreover, mutation of the mdv1-mir-M4-5p seed region within the TLR3 ORF abrogated the repressive effect of the miRNA and thus demonstrated the specificity of the target sequence for TLR3 mRNA (Fig 2c).

To test the effect of gga-miR-155 and mdv1-miR-M4 on regulating the endogenous TLR3 the CEF cells were transfected with a gga-miR-155 mimic or a mdv1-miR-M4-5p mimic, and the cells were cultured for 48 hours. Western blotting revealed that the TLR3 protein expression was decreased in the cells transfected with the gga-miR-155 mimic or with the mdv1-miR-M4-5p mimic (Fig 2d and 2e). Moreover, such inhibitory effects on TLR3 conferred by gga-miR-155 mimic or mdv1-miR-M4-5p mimic showed strong dose-dependence. All these results demonstrate that miR-155 can decrease TLR3 expression.

### TLR3 expression is regulated by miR-155

**Fig 3:**
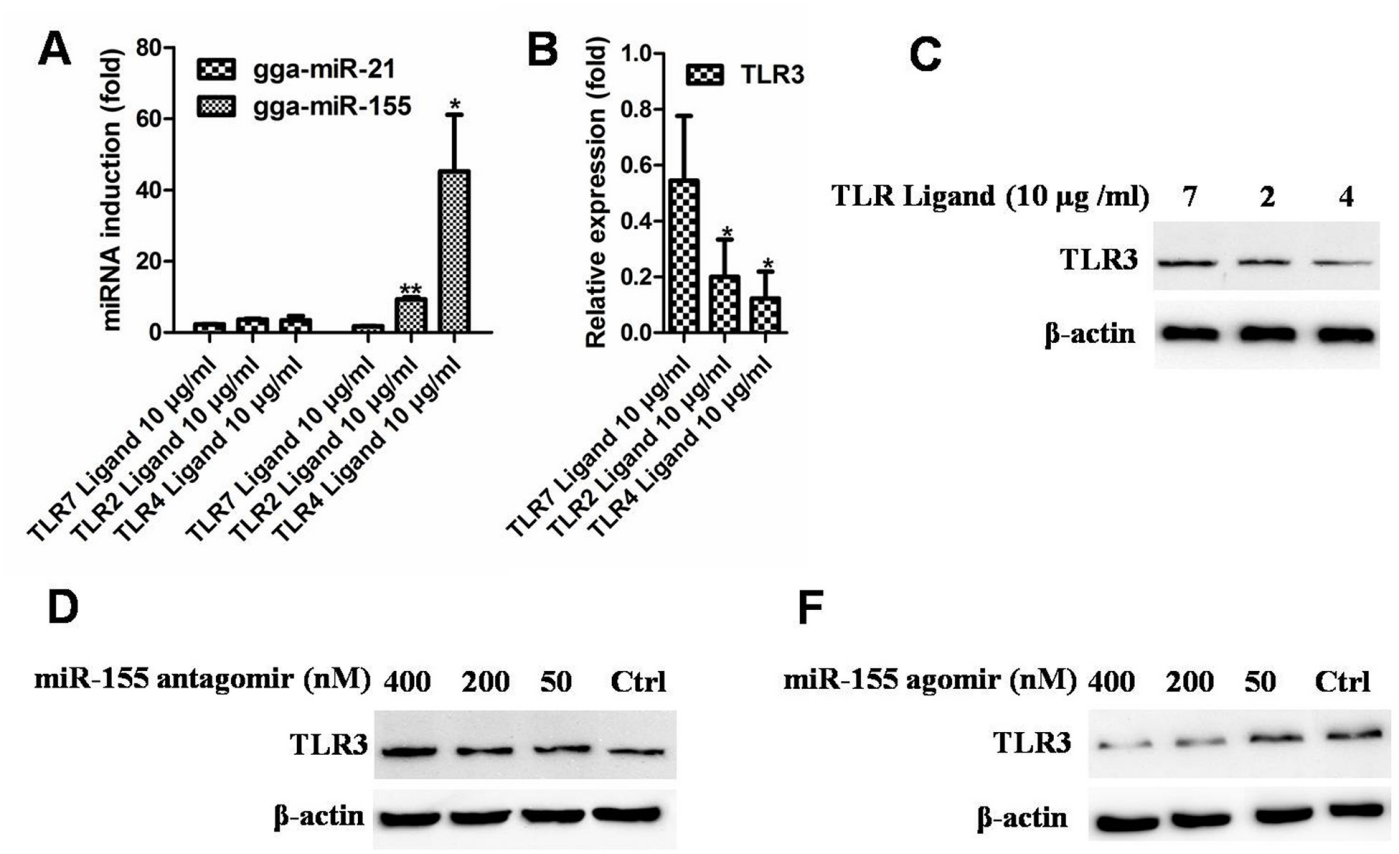
The regulation of TLR3 expression by miR-155. (a) miR-155 expression in HD11 cells treated with TLR2, 4 and 7 ligand. miR-21 expression was used as the control. (b) The relative expression (fold) of TLR3 mRNA expression in HD11 cells treated with TLR2, 4 and 7 ligand. Control group = 1 (c) The expression of TLR3 protein expression in HD11 cells treated with TLR2, 4 and 7 ligand. (d) The up-regulation of TLR3 protein expression in HD11 cells treated with 50–400 nM of the gga-miR-155 antagomir. (e) The down-regulation of TLR3 protein expression in HD11 cells treated with 50–400 nM of the gga-miR-155 agomir.

We next investigated whether there is the negative regulation relationship between TLR3 and endogenous miR-155. In TLR ligands treated HD11 cells (an avian macrophage cell line), TLR2 and TLR4 Ligands stimulation resulted in higher expression of gga-miR-155 at 24h while had little effect on the amount of gga-miR-21 (Fig 3a and 3b). Notably, we found miR-155 induction emerged a negative regulation on TLR3 mRNA expression (Fig 3b). Western blotting also showed that TLR3 protein expression was gradually decreased in HD11 cells along with the increased induction of miR-155 (Fig 3c).

To confirm the regulation of miR-155 on TLR3 expression, HD11 cells were treated for 48 h with increasing amounts of the miR-155 antagomir, which can inhibit miR-155 function. The treatment with the miR-155 antagomir resulted in increase TLR3 levels, and the concentrations of 400 nM were particularly effective (Fig 3d). In contrast, we treated HD11 cells with increasing amounts of miR-155 agomir significantly decreased the abundance of TLR3 (Fig 3e). These results suggest that TLR3 expression is regulated by miR-155.

### Regulation of IFN-β production by miR-155

**Fig 4:**
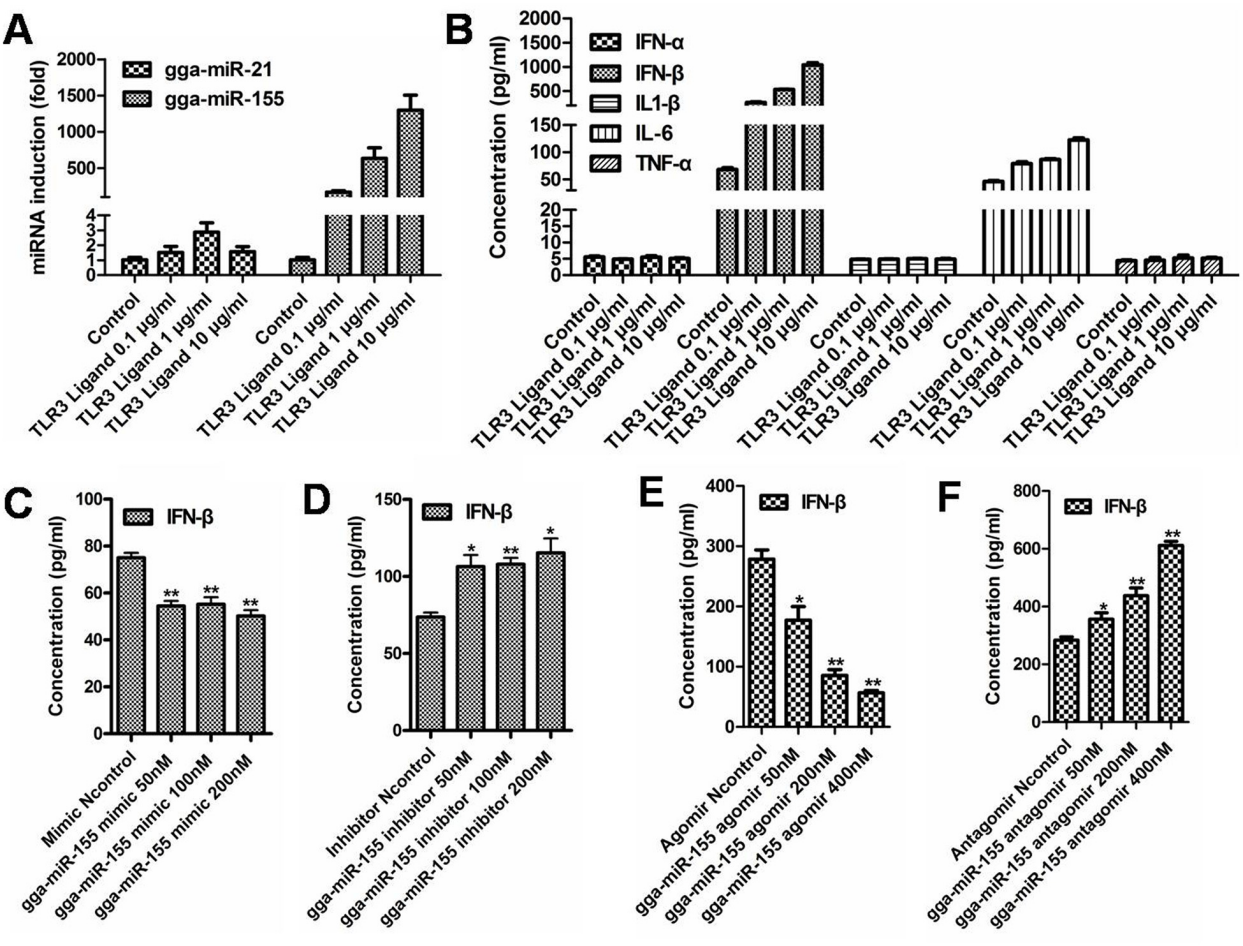
The regulation of IFN-β production by miR-155. (a) miR-155 expression in CEF cells treated with TLR3 ligand. miR-21 expression was used as the control. (b) ELISA for the productions of IFN-α, IFN-β, IL1-β, IL6 and TNF-α in the CEF cells treated with TLR ligands. (c) Down-regulation of IFN-β in CEF cells transfected with 50–200 nM of the gga-miR-155 mimic. (d) Up-regulation of IFN-β in CEF cells transfected with 50–200 nM of the gga-miR-155 inhibitor. (e) Down-regulation of IFN-β in HD11 cells treated with 50–400 nM of the gga-miR-155 agomir. (f) Up-regulation of of IFN-β in HD11 cells treated with 50–400 nM of the gga-miR-155 antagomir.

In CEF cells, we observed that poly (I:C) stimulation had no effect on the expression of miR-21,but it could upregulate the expression of miR-155 with dose dependence (from 0.1 μ g/ml to 10 μ g/ml, Fig 4a) at 24 h after stimulation. We also observed that the induction of miR-155 by poly (I:C) treatment was closely related to the IFN-β production in CEF cells (Fig 4b). As described in Fig 4c, IFN-β production was decreased with the treatment of the miR-155 mimic from 50–200 nM (Fig 4c). In contrast, IFN-β production was increased with the treatment of miR-155 inhibitor (Fig 4d). The consistent results were also observed in HD11 cells treated with increasing amounts of miR-155 agomir or antagomir (Fig 4e and 4f).

## Discussion

In the present study, we identified the existence of many naturally occurring miR-155 targets in the amino acid coding sequence of TLR3 and the essential role of miR-155 in the control of TLR3 expression. The expression of TLR3 was regulated by miR-155 and its functional orthologues. Our study demonstrated the up-regulation of miR-155 by poly (I:C) in chicken embryo fibroblast cells. *In vivo*, the up-regulation of miR-155 is closely related with a variety of disease (for review see[26]). The over-expression of miR-155 has been reported in many malignant tumours, such as Hodgkin's lymphoma. *In vitro*, the up-regulations of miR-155 following stimulation with toll-like receptor ligands (LPS, poly IC), IFN-β, IL-1β, TNF-α [27–29] and miR-155 have been shown to be a component of the primary macrophage responses to different types of inflammatory mediators. The correlation between miR-155 and the responses induced by LPS are consistent with the finding that miR-155 targets transcripts that code for proteins involved in LPS/TNF-α signalling, including FADD, IKKɛ and RIPK1 [8, 9] Additionally, miR-155 has been shown to regulate STAT1, which is the key controller of the interferon (IFN) network. Conversely, STAT1 also regulates miR-155. Recent findings support the presence of a positive feedback loop involving miR-155 and STAT1. in the response to inflammatory signals or infection [30]. Therefore, miR-155 might be an indicator of inflammation and an important link between cancer and inflammation.

The roles of miR-155 in the biologies and pathogeneses of several herpes viruses have been demonstrated. Studies have shown that the induction of miR-155 plays a key role in B-cell immortalisation by the human herpes virus (Epstein-Barr virus) through the NF-кB pathway [31, 32]. Two other oncogenic herpes viruses, Kaposi’s sarcoma-associated herpes virus and Marek’s disease virus, encode functional orthologues of miR-155 that are called miR-K12-11 (KSHV)[21, 33] and miR-M4 (MDV)[23, 34], respectively, and share the seed sequence of miR-155. The MDV-encoded miRNA miR-M4 has been shown to have an essential role in the induction of MD lymphoma [34]. Expression analysis of miR-M4 confirmed its high level of expression in MD tumours, and it is not possible to transform infected T-cells with a miR-M4 null mutant RB1B MDV-1 virus [34]. Here, we found that miR-M4 directly regulated TLR3 expression by targeting the TLR3 coding region. Alternatively, herpes viruses use the functional orthologues of miR-155 to alter host cell responses and/or promote their life cycles by targeting viral transcripts, and these seem to be the predominant mechanisms of the establishment of viral latency and the induction of lymphomas.

Currently, the relationships between miR-155 and TLR3 have been extensively studied in virus infection, immune response and tumour formation. TLR3 strongly enhances antigen-specific CD8+T-cell responses and promotes antigen cross-priming against virally infected cells [35–37]. miR-155 in CD8+ T cells is critical for generating CD8+ T cell responses against viral infection and exerts this control by regulating type I interferon signalling[18–20]. The expression of miR-155 has been implicated in lymphoid malignancies and is mediated by several oncogenic herpes viruses[21–23] to viral tumourigenesis, while the activation of TLR3 is involved in tumour suppression[5, 38, 39] via the recruiting of an anti-tumoural immune response. The available experimental evidence indicates that miR-155 is an important controller of TLR3 signals via the targeting of the downstream regulators, such as TAB2. Here, we demonstrated the existence of many naturally occurring miR-155 targets in the coding sequence (CDS) of TLR3. The expression of TLR3 was regulated by miR-155 and its functional orthologues.

The role of miR-155 in the TLR3 response is very complex: miR-155 expression not only exerts negative effects on the immune response and inflammation, but also acts as a positive regulator of immunity via the modulation of cytokine expression (for review see [8, 9]). An acceptable viewpoint is that miR-155 might function as a ‘brake’ or a molecular controller that represses the over-activation of the pro-inflammatory response without completely suppressing it. Here, we propose a viewpoint in which miR-155 could regulate TLR3 expression according to signalling thresholds rather than acting as an ‘off switch’ that intensively targets the TLR3 gene. Low levels of miR-155 expression would not trigger a negative regulation effect. In contrast, high expression levels of miR-155 would inhibit TLR3 signals, and decrease the expressions of TLR3 and other important signal transduction molecules (e.g., TAB2). Thus, through targeting the TLR3 signalling pathway, miR-155 might exert a regulatory activity that limits the over-production of type I interferon and inflammatory cytokines during viral infection and other stimuli.

A major function of TLR3 is to sense and respond to viral infection. TLR3 immunity appears to be a potent threat to viral infections and plays critical roles in protection against virus encephalitis (such as HSV and the West Nile virus) [40–43]. Clinical data suggest that patients with defects in the *TLR3/IFN* axis are much more susceptible to HSV-1 encephalitis[40, 41]. Thus, the control of TLR3 immunity is critical in viral infection. Interestingly, several studies have indicated that the tumour suppressor p53 positively regulates the transcription of *TLR3* by binding to the p53 site in the *TLR3* promoter and that TLR3 induction is p53-dependent in several tumour cell lines[44, 45]. Here, we found that TLR3 expression was negatively regulated by miR-155, and this could be suggested that the TLR3 signal was controlled by miR-155 over-expression.

TLR3 is a key player in antiviral immunity and tumour suppression and must be tightly regulated to avoid excessive inflammation and immune response. This receptor has been linked to tumour suppression via its recruitment of an anti-tumoural immune response or other unknown mechanisms. Activation of the TLR3 pathway by poly (I:C) has been reported to regulate IFN-β production in the chicken [46], and the induction of miR-155 by IFN-β has been demonstrated in mice [47]. Here, we found that the coding regions of TLR3 from different species (e.g., Homo sapiens and Gallus gallus) have binding sites for miR-155, which indicates that it is possible that miR-155 plays roles in the post-transcriptional regulation of *TLR3* mRNA. Indeed, miR-155 has been as a fine-tuner of TLR signalling pathways, and regulation by miR-155 may occur at various levels of the TLR pathways via the targeting of adaptor molecules, downstream regulators and cytokines (for review see [8, 9]). We further demonstrated that miR-155 directly regulated TLR3 expression by binding to its coding region, which also regulated IFN-β production.

The important mechanism by which TLR expression is directly regulated by miRNA has been uncovered. Currently, a few studies have indicated that TLRs themselves are directly targeted by miRNAs, and these interactions include the targeting of *TLR2* by miR-105[11] and miR-19[48], the targeting of *TLR4* by let-7i [10] and the targeting of *TLR7* by miR-3148[49]. Unlike these interactions, we found that miR-155 regulated the expression of TLR3 by targeting its coding region and not the 3’ UTR. Several miRNAs that target sites in the open reading frames (ORFs) of genes have also been observed in humans[50, 51], mice[24], and drosophila[52], but the extent of the biologically relevant targeting of the ORFs of the mRNAs of TLRs remains unknown. Our study is the first to reveal that miR-155, and particularly the viral encoded miR-155 orthologue, can regulate TLR3 expression. Although our findings have identified a previously unknown mechanism by which miR-155 can inhibit TLR3 signals, further studies should address the relevance of these findings in humans and mice to enhance our understanding of the regulatory functions of microRNA.
